# Validity of the RAI-MDS for ascertaining diabetes and comorbid conditions in long-term care facility residents

**DOI:** 10.1186/1472-6963-14-17

**Published:** 2014-01-15

**Authors:** Lisa M Lix, Lin Yan, David Blackburn, Nianping Hu, Verena Schneider-Lindner, Gary F Teare

**Affiliations:** 1 University of Manitoba, Winnipeg, MB, Canada; 2 University of Saskatchewan, Saskatoon, SK, Canada; 3 Health Quality Council, Saskatoon, SK, Canada; 4 Medical Faculty Mannheim, Heidelberg University, Heidelberg, Germany

**Keywords:** Chronic disease, Diagnostic validity, Long-term care, Nursing homes

## Abstract

**Background:**

This study assessed the validity of the Resident Assessment Instrument Minimum Data Set (RAI-MDS) Version 2.0 for diagnoses of diabetes and comorbid conditions in residents of long-term care facilities (LTCFs).

**Methods:**

Hospital inpatient, outpatient physician billing, RAI-MDS, and population registry data for 1997 to 2011 from Saskatchewan, Canada were used to ascertain cases of diabetes and 12 comorbid conditions. Prevalence estimates were calculated for both RAI-MDS and administrative health data. Sensitivity, specificity, and positive and negative predictive values (PPV and NPV) were calculated using population-based administrative health data as the validation data source. Cohen’s *κ* was used to estimate agreement between the two data sources.

**Results:**

23,217 LTCF residents were in the diabetes case ascertainment cohort. Diabetes prevalence was 25.3% in administrative health data and 21.9% in RAI-MDS data. Overall sensitivity of a RAI-MDS diabetes diagnoses was 0.79 (95% CI: 0.79, 0.80) and the PPV was 0.92 (95% CI: 0.91, 0.92), when compared to administrative health data. Sensitivity of the RAI-MDS for ascertaining comorbid conditions ranged from 0.21 for osteoporosis to 0.92 for multiple sclerosis; specificity was high for most conditions.

**Conclusions:**

RAI-MDS clinical assessment data are sensitive to ascertain diabetes cases in LTCF populations when compared to administrative health data. For many comorbid conditions, RAI-MDS data have low validity when compared to administrative data. Risk-adjustment measures based on these comorbidities might not produce consistent results for RAI-MDS and administrative health data, which could affect the conclusions of studies about health outcomes and quality of care across facilities.

## Background

Diabetes affects a high proportion of older adults and confers an increased risk for such conditions as hypertension, myocardial infarction, and stroke. Long-term care facilities (LTCFs), which provide housing, support, and nursing care for individuals who are no longer able to function independently, include a large and increasing number of residents with diabetes. The estimated prevalence of diabetes is much higher in LTCF populations than in the general population [1]. A 2002 US study reported a diabetes prevalence of 26.4% in LTCF residents, which was more than 30% higher than the estimate for the population 60+ years [2]. A more recent study estimated the prevalence to be even higher, at 32.8% [3].

Given the heavy demands that residents with diabetes can place on LTCF resources, information about diabetes is important for monitoring health outcomes and quality of care in LTCFs. As well, risk-adjustment methods, which are constructed using data about comorbid conditions, are essential to ensure fair comparisons across facilities or resident sub-populations when measuring outcomes.

Electronic, population-based data sources for ascertaining chronic and acute conditions in LTCFs include administrative health data, like hospital and physician records, and clinical assessment data, like the Resident Assessment Instrument Minimum Data Set (RAI-MDS) [4-6]. To date, comparisons of disease diagnostic information captured in these two data sources have been limited. Mor et al. [6] found that diagnoses in RAI-MDS data had fair to good sensitivity and specificity but low positive predictive value (PPV) when compared to diagnoses in hospital records for residents admitted to LTCFs from acute care hospitals. Wodchis et al. [7] obtained similar results. However, neither of these studies assessed validity of the diagnoses captured in the RAI-MDS for the entire LTCF resident population, not just those admitted from hospital, nor did they use both inpatient and outpatient data as the validation data source when assessing the validity of the RAI-MDS data.

The primary purpose of this study was to investigate the validity of diabetes diagnosis information recorded in population-based RAI-MDS. The secondary purpose was to investigate the validity of RAI-MDS diagnoses for comorbid conditions amongst LTCF residents with a diabetes diagnosis. Administrative health data were the validation source for both analyses.

## Methods

### Data sources

Population-based data from the province of Saskatchewan, Canada, which has a population of approximately 1.1 million according to the 2011 Statistics Canada Census, was used to conduct this study. Like all Canadian provinces, Saskatchewan has a universal health care program. The provincial ministry of health maintains health care databases in electronic format and these can be anonymously linked via a unique personal health number [8].

RAI-MDS Version 2.0, hospital inpatient records, outpatient physician billing claims, population registry records, and records from the provincial Institutional Supportive Care Home (ISCH) database were used to conduct the study. The RAI-MDS, originally developed by researchers under contract with the US Centers for Medicare and Medicaid Services, contains information about care and functioning of LTCF residents. This includes diagnoses for conditions that may affect activities of daily living, cognitive status, mood and behavior status, medical treatments, monitoring, and/or mortality risk. The data are collected via observations and documentation by LTCF staff and/or by interview with residents, relatives or doctors providing care to residents. Forms are required to be completed within 14 days of admission to a LTCF, as well as quarterly and annually thereafter, as well as whenever there is a major change in a resident’s health status. Saskatchewan was the first Canadian province to make the RAI-MDS mandatory in all LTCFs, in April 2001, although full implementation did not occur until 2004. RAI-MDS data from fiscal year 2005/06 (a fiscal year extends from April 1 to March 31) to 2011/12 (the most recent year of available data) were used in this study.

A hospital record is completed when a patient is discharged from an acute care facility. Diagnoses in hospital data are captured using ICD-9 up to and including the 2000/01 fiscal year. Beginning in 2001/02, diagnoses are recorded using ICD-10-CA. Between three and 16 diagnoses are captured in the data prior to the introduction of ICD-10-CA and up to 25 diagnoses are captured subsequently. Physicians paid on a fee-for-service basis submit billing claims to the ministry of health for payment purposes. A single diagnosis is recorded on each claim using ICD-9 codes. Some physicians, particularly specialists, are salaried and may not consistently submit billing claims. Hospital records and physician billing claims for fiscal years 1997/98 to 2011/12 were available.

The population registry contains dates of health insurance coverage, demographic information such as date of birth and sex, and location of residence. Registry data for fiscal years 1997/98 to 2011/12 were available.

The ISCH database contains information on new admissions, discharges, or changes in level of care classification for residents of LTCFs. The database also contains information on the characteristics of facilities.

The quality of Saskatchewan’s administrative health data for research has been documented in multiple studies [9-12]. Ethics approval for database access was received from the University of Saskatchewan Biomedical Research Ethics Board. Data were accessed and analyzed at the Health Quality Council in accordance with a standing data sharing agreement between the organization and the provincial health ministry.

### Study design

This study adopted a retrospective cohort design. The inclusion criteria for the diabetes case ascertainment cohort were: (a) an RAI-MDS admission or annual assessment between April 1, 2005 and March 31, 2011, and (b) continuous health insurance coverage between April 1, 1997 and the study index date. RAI-MDS quarterly assessments were not used to define the cohort because they lack complete information on diagnoses. The index date was the date that a diabetes diagnosis first appeared in an RAI-MDS admission or annual assessment between April 1, 2005 and March 31, 2011. If a person did not have a diabetes diagnosis in the RAI-MDS data during this time period, then the date of the first RAI-MDS admission or annual assessment between April 1, 2005 and March 31, 2011 was the index date.

The comorbidity case ascertainment cohort was a subset of the diabetes case ascertainment cohort. The inclusion criteria were: (a) diagnosis of diabetes in either RAI-MDS or administrative health data, and (b) at least 365 days of continuous health insurance coverage after the study index date, which is the same date as defined for the diabetes case ascertainment cohort. This coverage period was selected to allow for the potential diagnosis of comorbid conditions following a diabetes diagnosis.

### Study variables

Diagnosis information for diabetes and comorbid conditions was extracted from the RAI-MDS, hospital records, and physician billing claims. Socio-demographic variables were defined using the registry data. Information about LTCF characteristics was obtained from the ISCH data.

Diabetes cases were identified from the RAI-MDS data by searching for the first occurrence of a diabetes diagnosis in admission and annual assessments between April 1, 2005 and March 31, 2011. Diabetes cases from administrative health data were individuals who met a validated case definition for diabetes in hospital records or physician billing claims between April 1, 1997 and the study index date, inclusive (Table 1).

**Table 1:** Case definitions applied to administrative health data for diabetes and comorbid conditions

**Condition**	**Case definition algorithm**
Diabetes [13,14]	One or more inpatient hospital separations with an ICD code in any diagnosis field or two or more physician billing claims within two years with an ICD code.
	ICD-9: 250
	ICD-10-CA: E10, E11, E12, E13, E14
Alzheimer’s disease/dementia [15]	One or more inpatient hospital separations with an ICD code in any diagnosis field or one or more physician billing claims with an ICD code.
	ICD-9: 290, 291, 294, 331
	ICD-10-CA: F00, F01, F02, F03, F05.1, G30, G31.1
Arthritis [16,17]	One or more inpatient hospital separations with an ICD code in any diagnosis field or one or more physician billing claims with an ICD code.
	ICD-9: 714, 715, 446, 710, 720, 274, 711, 712, 713, 716,
	717, 718 719, 721, 725 to 729, 739
	ICD-10-CA: M05, M06, M15 to M19, M07, M10, M11 to M14, M30 to M36, M00 to M03, M20 to M25, M65 to M79
Cardiac dysrhythmia [17]	One or more inpatient hospital separations with an ICD code in any diagnosis field or one or more physician billing claims with an ICD code.
	ICD-9: 427
	ICD-10-CA: I47, I48, I49
Congestive heart failure [18,19]	One or more inpatient hospital separations with an ICD code in any diagnosis field or one or more physician billing claims with an ICD code.
	ICD-9 code: 428
	ICD-10-CA: I50
Chronic obstructive pulmonary disease [18]	One or more inpatient hospital separations with an ICD code in any diagnostic field or one or more physician billing claims with an ICD code.
	ICD-9 code: 491, 492, 496
	ICD-10-CA: J41, J42, J43, J44
Glaucoma [18]	One or more inpatient hospital separations with an ICD code in any diagnosis field or one or more physician claims with an ICD code.
	ICD-9 code: 365
	ICD-10-CA: H40, H42
Hip fracture [20]	One or more inpatient hospital separations with an ICD code in the first diagnosis field.
	ICD-9: 820
	ICD-10-CA: S72.0, S72.1, S72.2
Hypertension [21,22]	One or more inpatient hospital separations with an ICD code in any diagnosis field or two or more physician claims with an ICD code within two years.
	ICD-9: 401 to 405
	ICD-10-CA: I10 to I13, I15
Multiple sclerosis [15]	One or more inpatient hospital separations with an ICD code in any diagnosis field or one or more physician billing claims with an ICD code.
	ICD-9: 340
	ICD-10-CA: G35
Osteoporosis [23,24]	One or more inpatient hospital separations with an ICD code in any diagnosis field or one or more physician billing claims with an ICD code.
	ICD-9: 733.0 (hospital data) or 733 (physician data)
	ICD-10-CA: M80, M81
Parkinson’s disease [15]	One or more inpatient hospital separations with an ICD code in any diagnosis field or one or more physician billing claims with an ICD code.
	ICD-9: 332
	ICD-10-CA: G20
Stroke/transient ischemic attack [25]	One or more inpatient hospital separations with an ICD code in the first diagnosis field.
	ICD-9: 430, 431, 432, 433, 434, 435, 436, 437, or 438
	ICD-10-CA: I60, I61, I62, I63 (excluding I63.6), I64, I65, I66, I67 (excluding I67.6), I68, I69, G45, G46, H34.

Note: ICD-9 codes are used to ascertain cases in physician billing claims and in hospital records up to and including 2000/01 fiscal year; ICD-10-CA codes are used to ascertain cases in hospital records for 2001/02 onward and ICD-9 codes are used to ascertain cases in physician billing claims for 2001/02 onward.

We selected 12 comorbid conditions for which a validated case definition had been developed using diagnoses in hospital records and/or physician billing claims (Table 1), and for which diagnostic information was also available in the RAI-MDS Version 2.0: Alzheimer’s disease or dementia, arthritis, cardiac dysrhythmia, congestive heart failure, chronic obstructive pulmonary disease, glaucoma, hip fracture, hypertension, multiple sclerosis, osteoporosis, Parkinson’s disease, and stroke or transient ischemic attack. In the administrative data, stroke was the only condition defined exclusively from hospital records, while all other conditions were defined from both hospital records and physician billing claims. For all comorbid conditions, the observation window for case ascertainment extended from 365 days before to 365 days after the study index date in both the RAI-MDS and administrative health data.

Socio-demographic variables included age, sex, region of residence, and income quintile. All variables were defined at the study index date, except for income quintile, which was defined from the residence location one year prior to the index date. Age was classified as less than 65 years, 65 to 74 years, 75 to 84 years, and 85+ years. Rural and urban region of residence was based on postal code; a resident was categorized as living in an urban area if his/her postal code was in a Census Metropolitan Area or Census Agglomeration Area with a population of 10,000 or more. Income quintile was defined using a method based on average household income for Statistics Canada Census dissemination areas (DAs) [26]. DAs are the smallest geographic unit for which Census data are reported; each individual was assigned a DA based on his/her postal code; the highest quintile was the reference.

The Charlson comorbidity index score was computed for each resident. It was based on ICD diagnoses captured in hospital records and physician billing claims for the one-year period prior to the study index date and categorized as 0, 1 to 2, and 3 or more.

Facility type and affiliation were defined at the study index date. LTCFs were classified as special care home or other facility. The former are public facilities for which residence is determined based on need, while the latter are typically private facilities. LTCF affiliation includes amalgamate, affiliate, and contract. Health regions in the province may operate facilities on their own, or the facility may be operated by an independent health care organisation, or through a contract for services with an independent organization.

### Statistical analysis

Frequencies, percentages, means, and standard deviations (SDs) were used to describe the study cohort on socio-demographic and Charlson comorbidity variables. Crude prevalence estimates (percentages) were calculated for disease diagnoses in both data sources.

Sensitivity and specificity were estimated for diagnoses recorded in RAI-MDS data. Sensitivity was defined as the proportion of true positives that RAI-MDS detected among all positive disease cases. Positive disease cases were LTCF residents identified as having the disease in administrative health data based on the validated case definition for the disease. Specificity was defined as the proportion of true negatives the RAI-MDS detected among all negative disease cases. Negative disease cases were LTCF residents identified as not having the disease in administrative health data based on the validated case definition for the disease.

Positive predictive value (PPV) and negative predictive value (NPV) were also estimated for the RAI-MDS diagnoses. PPV refers to the proportion of LTCF residents with a RAI-MDS diagnosis for a specific disease among all LTCF residents who had the disease diagnosis. NPV refers to the proportion of LTCF residents without a RAI-MDS diagnosis amongst those residents who did not have the disease diagnosis.

Cohen’s *κ* was used to estimate agreement between the RAI-MDS and administrative health data. The interpretation of *κ* adopted in this study was [27]: *κ* < 0.20 is poor agreement, 0.20 ≤ *κ* ≤ 0.39 is fair agreement, 0.40 ≤ *κ* ≤ 0.59 is moderate agreement, 0.60 ≤ *κ* ≤ 0.79 is good agreement, and *κ* ≥ 0.80 is very good agreement.

For diabetes, overall estimates were produced for each of the indices of sensitivity, specificity, PPV, NPV, and *κ*. In addition, separate estimates were produced by age group (i.e., less than 65 years, 65 years and older), sex, and the type of RAI-MDS assessment (i.e., admission versus annual). For the comorbid conditions, only overall estimates were produced because there were too few cases of some conditions to conduct stratified analyses.

As well, 95% confidence intervals (CIs) were produced for all estimates; they were based on the asymptotic standard error and a critical value from the standard normal distribution. Analyses were conducted using SAS software, version 9.3. [28].

## Results

The study cohort for diabetes case ascertainment consisted of 23,217 LTCF residents with an average age of 83.4 years (SD = 11.4; median = 86 years). Close to two-thirds (63.7%) were female (Table 2), and there was a higher proportion of residents in the lowest (23.3%) than highest income quintile (13.5%). The cohort was almost equally split between rural and urban residents. For almost three-quarters (74.0%) of the cohort, the study index date corresponded to an RAI-MDS admission assessment.

**Table 2:** **Characteristics of long-term care facility residents in the diabetes case ascertainment cohort (*N*** **= 23,217) and comorbidity case ascertainment cohort (*N*** **= 4,183)**

**Variable**	**Diabetes case ascertainment cohort *n *** **(%)**	**Comorbidity case ascertainment cohort *n *** **(%)**
Age group		
< 65 years	1518 (6.5)	300 (7.2)
65 – 74 years	2090 (9.0)	554 (13.2)
75 – 84 years	7187 (31.0)	1517 (36.3)
85+ years	12422 (53.5)	1812 (43.3)
Sex		
Female	14795 (63.7)	2547 (60.9)
Male	8422 (36.3)	1636 (39.1)
Charlson comorbidity index		
0	7947 (34.2)	1108 (26.5)
1-2	9467 (40.8)	1552 (37.1)
3+	5803 (25.0)	1523 (36.4)
Income quintile		
Q1 (Lowest)	5400 (23.3)	1097 (26.2)
Q2	4814 (20.7)	887 (21.2)
Q3	5639 (24.3)	983 (23.5)
Q4	4072 (17.5)	693 (16.6)
Q5 (Highest)	3143 (13.5)	500 (12.0)
Missing	149 (0.6)	23 (0.5)
Residence location		
Rural	11796 (50.8)	2197 (52.5)
Urban	11209 (48.3)	1932 (46.2)
Missing	212 (0.9)	54 (1.3)
Facility affiliation		
Affiliated	6834 (29.4)	1160 (27.7)
Amalgamated	14412 (62.1)	2686 (64.2)
Contract	1968 (8.5)	334 (8.1)
Facility type		
Special care home	20002 (90.5)	3787 (90.5)
Other facility	2215 (9.5)	396 (9.5)
Diabetes prevalence (%)	6298 (27.1)	4183 (100.0)

**Figure 1:**
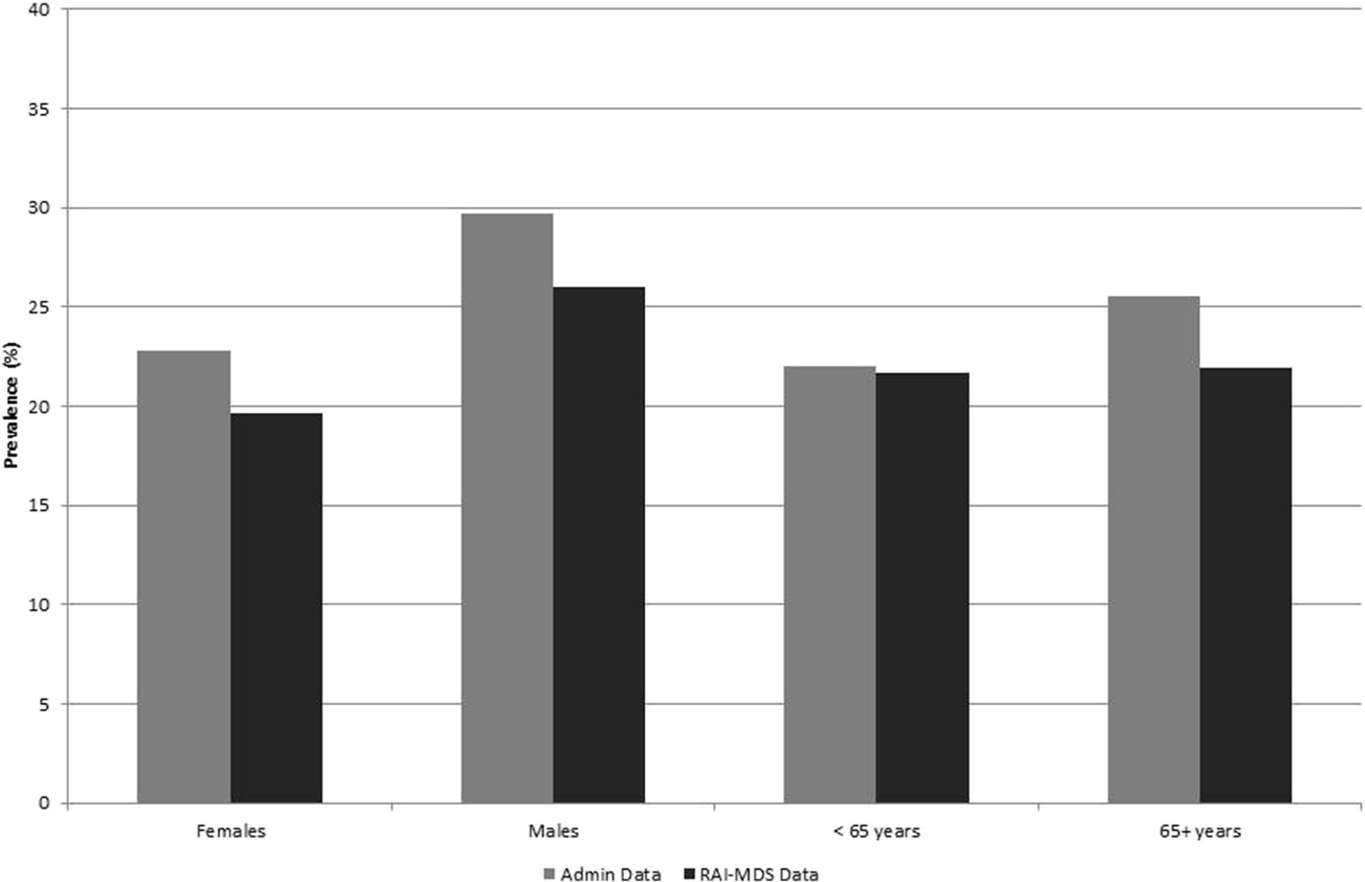
Prevalence of diagnosed diabetes in RAI-MDS data and administrative health data by sex and age group.

The prevalence of diabetes (Figure 1) was 27.1% for both data sources combined, with estimates of 25.3% for administrative health data and 21.9% for RAI-MDS data only. Prevalence was higher in males (32.1%) than females (24.8%) and was lower in those less than 65 years (25.9%) than in those 65+ years (38.1%). While the prevalence estimates from administrative health data were slightly higher than those from the RAI-MDS data by age and sex, there was very good agreement between the two data sources.

For the full cohort, sensitivity of a RAI-MDS diabetes (Table 3) was 0.79 (95% CI: 0.79, 0.80) and specificity was 0.97 (95% CI: 0.97, 0.98). PPV and NPV estimates were above 0.90. The estimate of Cohen’s *κ* was 0.81 (95% CI: 0.80, 0.82). Sensitivity, specificity, NPV, and *κ* were slightly higher in younger than older age groups, while the reverse was true for PPV. Sensitivity and specificity were similar when the index RAI-MDS assessment was an admission rather than an annual assessment. However, PPV was higher for the former than the latter, as was *κ*.

**Table 3:** **Estimates of validation and agreement measures for diagnosed diabetes in RAI-MDS data for the diabetes case ascertainment cohort (*N*** **= 23,217)**

	**Sens. (95% CI)**	**Spec. (95% CI)**	**PPV (95% CI)**	**NPV (95% CI)**	***κ *** **(95% CI)**
Full cohort	0.79 (0.79, 0.80)	0.98 (0.97, 0.98)	0.92 (0.91, 0.92)	0.93 (0.93, 0.94)	0.81 (0.80, 0.82)
Females	0.79 (0.78, 0.79)	0.98 (0.98, 0.98)	0.91 (0.91, 0.93)	0.94 (0.94, 0.94)	0.80 (0.79, 0.82)
Males	0.81 (0.80, 0.82)	0.97 (0.97, 0.97)	0.92 (0.92, 0.93)	0.92 (0.92, 0.93)	0.81 (0.79, 0.82)
< 65 years	0.86 (0.84, 0.88)	0.96 (0.96, 0.97)	0.87 (0.86, 0.89)	0.96 (0.95, 0.97)	0.83 (0.79, 0.86)
65+ years	0.79 (0.79, 0.80)	0.98 (0.97, 0.98)	0.92 (0.92, 0.92)	0.93 (0.93, 0.93)	0.80 (0.80, 0.81)
Admission RAI-MDS assessment	0.79 (0.79, 0.80)	0.98 (0.98, 0.98)	0.94 (0.93, 0.94)	0.93 (0.92, 0.93)	0.81 (0.80, 0.82)
Annual RAI-MDS assessment	0.80 (0.79, 0.81)	0.96 (0.96, 0.97)	0.85 (0.84, 0.86)	0.95 (0.94, 0.95)	0.78 (0.76, 0.80)

Sens. = sensitivity; Spec. = specificity; PPV = positive predictive value; NPV = negative predictive value; CI = confidence interval.

The comorbidity case ascertainment cohort, which was a subset of the diabetes case ascertainment cohort (*N* = 4,183), is described in Table 2. This cohort was slightly younger than the diabetes case ascertainment cohort, with a mean age of 81.5 years (SD = 10.5; median = 84), but had higher overall comorbidity.

As Table 4 reveals, the comorbid condition with the highest prevalence in both RAI-MDS and administrative health data was hypertension (55.8% and 57.3%, respectively), with more than half of the diabetes cohort having a diagnosis. Prevalence was almost equally high for arthritis in administrative health data (53.0%), but was substantially lower in RAI-MDS data (42.5%). Prevalence was higher in RAI-MDS than administrative data for one-third of the comorbid conditions, including Alzheimer’s disease or dementia, glaucoma, hip fracture, and stroke or TIA (Table 4), but higher in administrative health data than RAI-MDS data for the remainder of the conditions.

**Table 4:** **Estimates of prevalence, validation and agreement measures for comorbid conditions in RAI-MDS data for the comorbidity case ascertainment cohort (*N*** **= 4,183)**

**Condition**	**RAI-MDS Prev. (%)**	**Admin. data prev. (%)**	**Sens. (95% CI)**	**Spec. (95% CI)**	**PPV (95% CI)**	**NPV (95% CI)**	***κ *** **(95% CI)**
Hypertension	55.8	57.3	0.68 (0.67, 0.70)	0.61 (0.59, 0.62)	0.70 (0.69, 0.72)	0.59 (0.57, 0.60)	0.29 (0.26, 0.32)
Congestive heart failure	18.7	31.8	0.39 (0.37, 0.40)	0.91 (0.90, 0.92)	0.66 (0.65, 0.68)	0.76 (0.75, 0.77)	0.33 (0.30, 0.37)
Arthritis	42.5	53.0	0.51 (0.49, 0.52)	0.67 0.65, 0.68)	0.63 (0.62, 0.65)	0.55 (0.53, 0.56)	0.17 (0.14, 0.20)
COPD	9.6	13.8	0.44 (0.42, 0.45)	0.96 (0.95, 0.96)	0.63 (0.61, 0.64)	0.91 (0.91, 0.92)	0.45 (0.41, 0.49)
Multiple sclerosis	2.0	1.4	0.92 (0.91, 0.92)	0.99 (0.99, 1.00)	0.65 (0.64, 0.67)	1.00 (1.00, 1.00)	0.76 (0.68, 0.84)
Parkinson`s disease	5.0	5.9	0.61 (0.60, 0.63)	0.99 (0.98, 0.99)	0.72 (0.70, 0.73)	0.98 (0.97, 0.98)	0.64 (0.59, 0.69)
Cardiac dysrhythmia	11.1	26.7	0.27 (0.26, 0.28)	0.95 (0.94, 0.95)	0.65 (0.64, 0.67)	0.78 (0.77, 0.79)	0.27 (0.24, 0.30)
AD/dementia	44.2	31.9	0.71 (0.70, 0.73)	0.69 (0.67, 0.70)	0.52 (0.50, 0.53)	0.84 (0.83, 0.85)	0.36 (0.34, 0.39)
Glaucoma	7.6	7.2	0.53 (0.51, 0.54)	0.96 (0.95, 0.97)	0.50 (0.49, 0.52)	0.96 (0.96, 0.97)	0.48 (0.43, 0.53)
Osteoporosis	13.5	31.4	0.21 (0.20, 0.22)	0.90 (0.89, 0.91)	0.49 (0.47, 0.50)	0.71 (0.70, 0.73)	0.13 (0.10, 0.16)
Hip fracture	10.2	8.9	0.62 (0.60, 0.63)	0.95 (0.94, 0.96)	0.54 (0.52, 0.55)	0.96 (0.96, 0.97)	0.53 (0.49, 0.58)
Stroke/TIA	30.6	45.5	0.65 (0.63, 0.66)	0.81 (0.80, 0.82)	0.53 (0.52, 0.55)	0.87 (0.86, 0.88)	0.42 (0.39, 0.45)

Note: COPD = chronic obstructive pulmonary disease; AD = Alzheimer’s Disease; TIA = transient ischemic attack; Sens. = sensitivity; Spec. = specificity; PPV = positive predictive value; NPV = negative predictive value; CI = confidence interval.

Validity of the RAI-MDS diagnoses for the comorbid conditions varied substantially. Sensitivity was lowest for osteoporosis (0.21; 95% CI: 0.20, 0.22) and highest for multiple sclerosis (0.92; 95% CI: 0.91, 0.92). Specificity was below 0.90 for hypertension, arthritis, Alzheimer’s disease/dementia, and stroke/transient ischemic attack. PPV estimates ranged from 0.49 (95% CI: 0.47, 0.50) for osteoporosis to 0.72 (95% CI: 0.70, 0.73) for Parkinson’s disease. NPV estimates ranged from 0.55 (95% CI: 0.53, 0.56) for arthritis to 1.00 (95% CI: 1.00, 1.00) for multiple sclerosis. Estimates of *κ* did not exceed 0.60 for any the comorbid conditions except for multiple sclerosis (*κ* = 0.76) and Parkinson’s disease (*κ* = 0.64); agreement was very low for osteoporosis (*κ* = 0.13) and arthritis (*κ* = 0.17).

## Discussion

This study validated diabetes diagnoses captured in RAI-MDS data for LTCF residents and then further examined the validity of multiple comorbid conditions in residents with a diabetes diagnosis using administrative health data as the validation data source. The findings revealed good sensitivity and excellent specificity of the RAI-MDS for diabetes. PPV, NPV, and *κ* estimates were also very good. However, for 12 comorbid conditions, there was substantial variability in estimates of validity and agreement.

RAI-MDS data, while originally intended for clinical assessment, are now being used to measure quality of care and resident health outcomes across LTCFs, such as medication management and glycemic control in diabetics, [1] as well as outcomes of care such as falls, amputations, and skin ulcers [29]. To ensure these measures can be fairly compared across LTCFs, it is important to use risk-adjustment measures based on comorbid conditions, which account for underlying differences in resident populations. For example, Berlowitz et al. [30] developed a model for predicting pressure ulcers, in which resident comorbid conditions of diabetes and hip fracture were used for risk adjustment.

While previous studies have estimated diabetes prevalence in LTCF populations, [1,2] there has been limited data about the prevalence of other health conditions. Travis et al. [2] reported a hypertension prevalence of 69.1% in LTCF residents at admission, which is substantially higher than our estimates of 55.8% from RAI-MDS data and 57.2% from administrative health data. However, for cardiac dysrhythmia, our RAI-MDS estimate was similar (11.1%) to the estimate from this earlier study (12.4%).

Our study reveals both similarities and differences with previous research about the validity of diagnoses in RAI-MDS admission assessments for residents admitted from hospital when diagnoses captured in hospital records were used as the validation data source. Mor et al. [6] reported a sensitivity of 0.93 for diabetes, which is only slightly higher than our estimate. However, PPV was only 0.69 in that study, while our estimate exceeded 0.90. Wodchis et al. [7] reported sensitivity of RAI-MDS diagnoses ranging from 0.16 for hypotension to 0.91 for cancer. For Alzheimer’s disease, a common condition in LTCF residents, sensitivity was 0.64, while for other dementia it was 0.60. Both values are similar to the estimate of 0.71 that we observed for Alzheimer’s disease or dementia. Sensitivity of the RAI-MDS data for hypertension was also similar between the two studies. However, for osteoporosis, Wodchis et al. [7] reported a sensitivity estimate of 0.65, which is substantially higher than our estimate of 0.21.

While previous research has focused on the validity of disease information captured in the RAI-MDS admission assessment, the current study undertook validity comparisons of both admission and annual assessments for diabetes. Sensitivity estimates were similar between the two assessment sources, but PPV and *κ* estimates were higher in the admission assessment. This difference may reflect a lack of consistency checks amongst LTCF staff, to ensure that conditions present on admission continue to be recorded in follow-up assessments [31].

In Canada, the Canadian Institute for Health Information (CIHI), a national not-for-profit agency that develops and maintains comprehensive and integrated health information, compiles RAI-MDS data from LTCFs in its Continuing Care Reporting System (CCRS) for seven of the thirteen provinces and territories; the CCRS was established in 2003 [31]. More than 1000 facilities submitted data to the CCRS in the 2011/12 fiscal year, including all facilities in the province of Ontario and selected facilities in other provinces and territories. The CCRS maintains standards for data element specifications, valid code values, format of records, and data validation rules. Quality of the data submitted to the CCRS is evaluated annually. Saskatchewan currently submits its RAI-MDS data to CIHI, although not via the CCRS and the Saskatchewan data are therefore not included in national quality reports [32]. This may affect the generalizability of the current study findings to other Canadian provinces and territories.

There are several potential reasons for the differences between the RAI-MDS data and administrative health data for ascertaining cases of diabetes and comorbid conditions; both data sources have strengths and limitations. Conditions recorded in the RAI-MDS are limited to “active” conditions that affect resident activities of daily living, cognition, mood, or behavior. Conditions that are present, but that have minimal impact on resident day-to-day function, such as osteoporosis, are less likely to be recorded in the RAI-MDS data. A longer time frame than was used in the current study may be needed to capture information about comorbid conditions in the RAI-MDS. Some conditions such as osteoporosis may have a higher probability of capture in primary care data (i.e., physician claims) because ongoing treatment with prescription medications may result in multiple visits to physicians. Second, LTCF staff may not comprehensively record data in the assessment tool because of competing demands on their time; they may therefore focus on recording information about patient functional abilities rather than health conditions [7]. This too may account for the lower prevalence of several comorbid conditions in the RAI-MDS data. Staffing levels within facilities may affect the time available for LTCF staff to record assessment data. Residents may be admitted near the end of life, limiting the number of assessment opportunities to capture diagnostic information. Given that LTCF residents, particularly those with diabetes, are likely to have many outpatient and inpatient healthcare contacts to manage and treat their multiple health conditions, there may be more opportunities to capture disease diagnoses in administrative health data, although this may also result in an increased rate of false positive cases. For some high-prevalence conditions in LTCF residents such as Alzheimer’s disease/dementia, RAI-MDS data, despite having low sensitivity when compared to administrative health data, may have advantages over administrative health data for case ascertainment because LTCF staff may be more sensitive to the signs and symptoms of the disease than physicians.

This study has some limitations. We selected only a single case definition to apply to administrative health data for ascertaining cases of diabetes and comorbid conditions; different results may have been obtained with case definitions having different magnitudes of sensitivity and specificity. As well, estimates of validity will be affected by the choice of validation data sources; different estimates may have been obtained using the medical chart(s) of patients as the validation data source. We did not include all chronic conditions for which data are available in the RAI-MDS, instead limiting attention to conditions for which a validated algorithm existed for administrative health data. Finally, while our study results highlight some issues with the validity of disease diagnosis information in the RAI-MDS, they do not suggest how validity may be improved.

## Conclusions

In summary, this study found that validity of diagnostic information in RAI-MDS was very good for diabetes but variable, and generally poorer, for comorbid chronic conditions when administrative health data were used as the validation data source. Based on the study results, we recommend that researchers can use either RAI-MDS or administrative health data to define population-based cohorts of LTCF residents with diabetes for observational studies; either data source will produce comparable results. However, for risk adjustment, we recommend using linked hospital records and physician billing claims because these data are more likely to capture a broad range of comorbid conditions. Erring on the side of caution by including as many comorbid conditions as may likely be present in LTCF residents is wise, given that unmeasured confounding can attenuate the association between disease status and health outcomes [33].

## Competing interests

The authors declare that they have no competing interests.

## Authors’ contributions

LML, LY, VSL, DB, and GFT conceived the study. LY and NH conducted the data extraction and carried out the statistical analyses. LML, LY, and NH summarized the data. LML, LY, DB, VSL, and GFT drafted the manuscript. All authors read and approved the final manuscript.

## Pre-publication history

The pre-publication history for this paper can be accessed here:

http://www.biomedcentral.com/1472-6963/14/17/prepub
